# Genome-Wide Identification and Expression Profiling of Velvet Complex Transcription Factors in *Populus alba* × *Populus glandulosa*

**DOI:** 10.3390/ijms25073926

**Published:** 2024-03-31

**Authors:** Yuanyuan Hao, Xiaojing Yan, Quanzi Li

**Affiliations:** State Key Laboratory of Tree Genetics and Breeding, Chinese Academy of Forestry, Beijing 100091, China; haoyuanyuan@caf.ac.cn (Y.H.); liqz@caf.ac.cn (Q.L.)

**Keywords:** PagVeA, PagMYB128, transcription factor, secondary metabolism, *P. alba* × *P. glandulosa*

## Abstract

The discovery of new genes with novel functions is a major driver of adaptive evolutionary innovation in plants. Especially in woody plants, due to genome expansion, new genes evolve to regulate the processes of growth and development. In this study, we characterized the unique *VeA* transcription factor family in *Populus alba* × *Populus glandulosa*, which is associated with secondary metabolism. Twenty VeA genes were characterized systematically on their phylogeny, genomic distribution, gene structure and conserved motif, promoter binding site, and expression profiling. Furthermore, through ChIP-qPCR, Y1H, and effector-reporter assays, it was demonstrated that PagMYB128 directly regulated *PagVeA3* to influence the biosynthesis of secondary metabolites. These results provide a basis for further elucidating the function of *VeAs* gene in poplar and its genetic regulation mechanism.

## 1. Introduction

Poplar is one of the most distributed woody plants in the world and has important ecological and economic value. As a fast-growing, high-yield, and widely used tree species, poplar plays an irreplaceable role in pulp and paper, bioenergy, wind and sand fixation, and industrial production. Because of its advantages such as fast growth, easy propagation, early timber, and easy transformation, it is currently the main model tree species for molecular biology research on woody plants [1]. Accurate genome sequence analysis and comprehensive and systematic gene function research can provide a basis for comparative genome analysis and molecular breeding practices of poplar. At present, the genome sequences of various poplar trees have been assembled and analyzed, such as *P. trichocarpa* [2,3], *P. alba* × *P. glandulosa* [4], *P. alba* [5], *P. tremula* [6], *P. euphratica* [7], *P. koreana* [8], *P. cathayana* [9], *P. euphratica*, and *P. alba var. pyramidalis* [9], etc. On this basis, the mechanisms of functional genes involved in various biological processes of poplar have also been studied, such as growth and development [10,11], stress response [12,13], wood formation [14,15,16], and sexual differentiation. However, as a woody plant, poplar has some unique features in terms of life cycle, morphological structure, and environmental adaptability [6,12]. Therefore, poplar may have some unique genes and gene families that differ from herbaceous plants in these aspects. 

Secondary metabolism refers to the process of synthesizing non-essential small molecule organic compounds (such as secondary metabolites) that is carried out in the body and does not directly meet the metabolic needs of the organism [17,18]. Secondary metabolites not only play important functions in plant growth and development, but also help plants adapt to adverse environments, such as resisting stress conditions such as drought, high salt, and low temperature [13,19,20]. Indole-3-acetic acid and gibberellins (GA) are directly involved in the regulation of plant life activities [21]. Anthocyanins have photoprotective properties, helping plants resist damage from UV rays and other harmful rays [22,23,24,25]. Secondary metabolites such as cellulose and lignin are important components of plant cell walls and help maintain cell integrity and stability [9,26,27]. Poplar trees have a secondary growth process in their stems that is unique to woody plants. During this process, the secondary xylem cell wall thickens to form a secondary wall, which is mainly composed of cellulose, hemicelluloses, lignin, and other important secondary metabolic substances, which determine the quality of wood [28,29,30,31]. Many studies have shown that members of the MYB transcription factor family are key factors in regulating the biosynthesis and accumulation of secondary metabolites in plants [25,32,33,34,35,36,37]. Genetic and molecular studies have shown that AtMYB103 plays an important role in the regulatory network of secondary metabolism in *Arabidopsis* [31]. Inhibition of *AtMYB103* will affect the synthesis of cell wall, cellulose content, and flavonoids content [38]. *MYB10* and *MYB128* are homologous genes of *AtMYB103* in *Populus*. In *P. deltoides Marshall*, overexpression of *PdMYB10* and *PdMYB128* resulted in thickening of secondary cell walls and increased lignin, cellulose, and hemicellulose content [29,38]. In *P. alba*, overexpression of *PtoMYB10* could inhibit anthocyanin accumulation [29]. Although studies have shown that they are related to the synthesis of secondary metabolites, there are few studies on their clear mechanism of action and upstream and downstream interacting proteins.

In order to identify the direct targets of PagMYB128 in *P. alba* × *P. glandulosa*, we conducted RNA-seq of *35S-PagMYB128* overexpression transgenic plants and ChIP-seq of *PagMYB128:FLAG* overexpression transgenic plants. Through correlation analysis of the sequencing results, we found *PagVeA3* (*Velvet complex transcription factor 3*) may be the downstream regulatory gene of *PagMYB128*. Through further sequence comparison (NCBI Blast), we found that *PagVeA3* only existed in *Salicaceae* plants and had no homologous sequence in Arabidopsis and other woody plants. Therefore, in this study, we conducted a systematic identification and analysis of VeA family members in *P. alba* × *P. glandulosa*. In addition, we verified the regulation of *PagVeA3* by *PagMYB128* through Y1H, Split-LUC, and ChIP-qPCR assays. Relevant results provide some new theoretical basis for the regulation of secondary metabolites in poplar.

## 2. Results

### 2.1. Genome-Wide Identification of VeA in P. alba × P. glandulosa

In order to conduct a comprehensive and systematic analysis of the *VeA* gene family, we used the obtained PagVeA3 (Pag_A_036054-RA) protein sequence as a conserved region and then aligned it to the *P. alba* × *P. glandulosa* genome to identify the putative *VeA* genes in poplar. Blast results identified a total of putative 20 *VeA* genes. Gene characterization assays showed that the ORF length of these *VeA* genes ranged from 990 to 2379 bp, and the amino acid length ranged from 329 to 792 amino acids. The average molecular weight (Mw) of these gene was 61.44 kDa, and the isoelectric point (PI) was 11.99 ~ 12.67. Subcellular localization prediction results showed that 20 genes were all located in the nucleus (Table 1).

Some studies have shown that VeA was a central player of the light regulatory network, and it is also involved in regulating production of secondary metabolites in *genus Aspergillus* [39]. To further demonstrate the specificity of the *PagVeA3* gene, we performed sequence alignment analysis with the VeA proteins in fungi [17,40] such as *Emericella nidulans*, *Aspergillus fumigatus*, *A. flavus*, *Fusarium oxysporum* f. sp. *Lycopersici*, *Hapsidospora chrysogena*, *A. niger*, etc. After aligning the protein sequences of PagVeA3 with those of typical VeA proteins in fungi, we found that the similarity between PagVeA3 and the typical VeA proteins in fungi was extremely low (Figure S1). Therefore, we ruled out the possibility that *PagVeA3* was an endophytic gene of poplar. Meanwhile, we compared and searched the PagVeA3 protein sequence in the NCBI database and found that this conserved region also exists in *P. alba* [5], which is a parent of *P. alba* × *P. glandulosa* (Figure S2). All the accession numbers for the VeA gene and protein sequence data are shown in Table S1.

### 2.2. Phylogenetic Analysis of VeA Genes in Poplar

**Figure 1:**
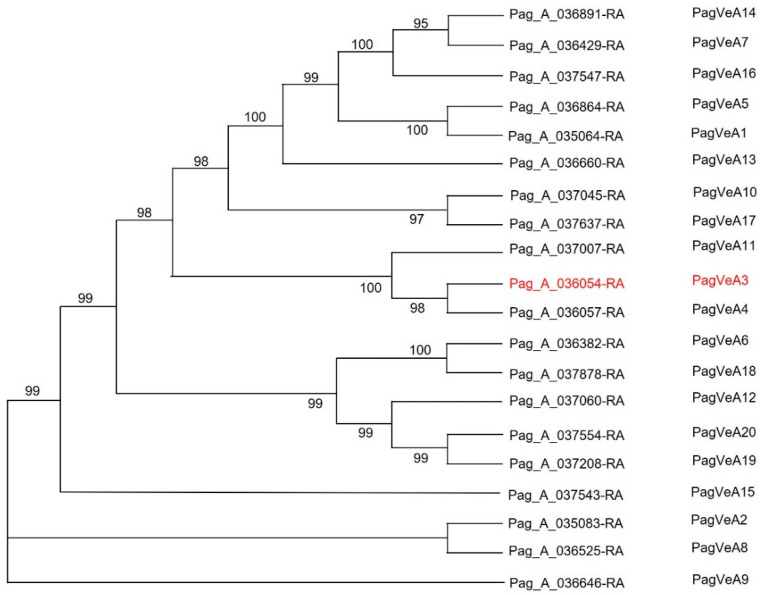
Phylogenetic analysis of VeAs in *P. alba* × *P. glandulosa*. The phylogenetic tree was constructed using MEGA 7.0 by the neighbor-joining (NJ) method with 1000 bootstrap replicates. PagVeA3 is shown in red.

To investigate the phylogenetic relationship of the *VeA* genes in poplar, we conducted the molecular phylogenetic analysis. As shown in the phylogenetic tree (Figure 1), PagVeA3 and PagVeA4 had 82% sequence similarity, E-value < 0.05, and evolutionary tree step value > 90. The results indicated that *PagVeA3* and *PagVeA4* were highly conserved in evolution. The bootstrap test results showed that the step values of *VeA* gene family were all greater than 60. The above analysis results suggested that the *VeA* gene family played a conservative role in *P. alba* × *P. glandulosa*. The names of these 20 genes are listed in Table S2.

### 2.3. Genomic Distribution Analysis of VeA Genes

**Figure 2:**
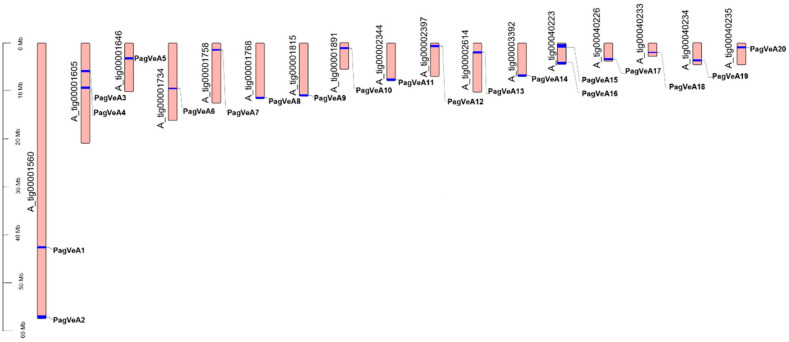
Genomic distribution analysis of *VeA* genes.

In order to further analyze the phylogenetic relationship of the *VeA* gene family members, we determined the genomic distribution location analysis of them. The results showed that these genes were distributed on 17 contigs (Figure 2). According to the position of these genes on the genome, we named *Pag_A_035064-RA* as *PagVeA1*, and the rest were named in numerical order from small to large. Among them, A_tig0001560, A_tig0001605, and A_tig00040223 contained two *VeA* genes, and the rest included one *VeA* gene, respectively. The genomic distribution location analysis results of these *VeA* gene family members can provide information for future VeA-based gene editing and genetic improvement of poplar.

### 2.4. Gene Structure and Conserved Motif Analysis of VeA Genes

**Figure 3:**
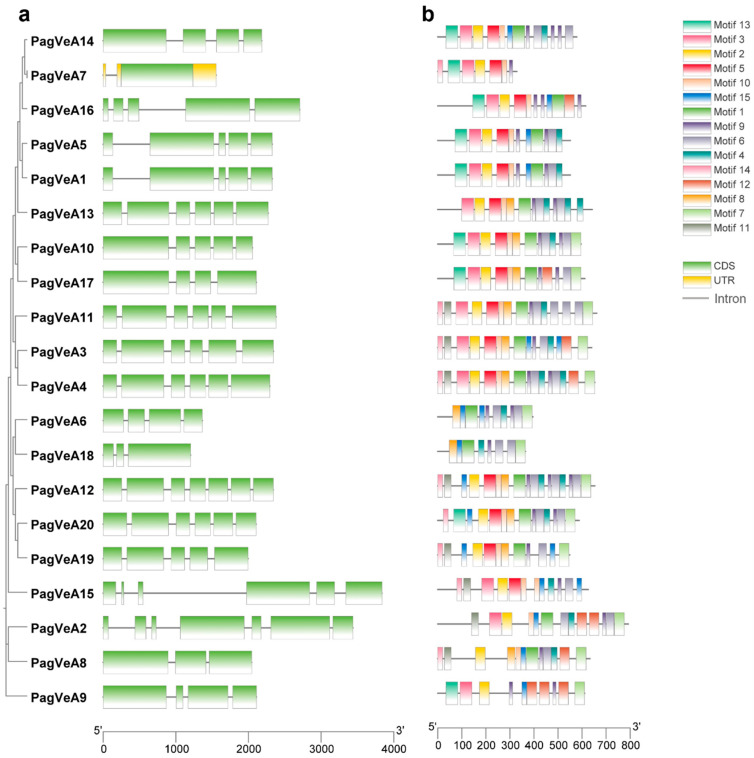
The conserved motifs and gene structure of *VeA* genes. Exons, introns, and UTRs are shown in (**a**); motifs are represented by boxes in (**b**).

The exon-intron pattern is an important feature of genes, which can provide important evidence for the diversification of gene functions. Therefore, we investigated the exon-intron patterns of the *VeA* gene family members. Among these *VeA* genes, except *PagVeA7* (*Pag_A_036429-RA*) which has only one exon, the rest contained exons and introns. The number of exons in *VeA* gene family ranged from 3 to 7, of which *PagVeA3* and *PagVeA4*, *PagVeA1*, and *PagVeA5* have similar gene structures (Figure 3a). The results showed that genes clustered in the same branch of the phylogenetic tree had similar exon-intron patterns. Further conserved domain analysis results also showed that genes clustered in the same branch had similar motifs. For example, *VeA3* and *VeA4* contained 14 identical motifs (Figure 3b).

### 2.5. Promoter Binding Site Analysis of VeA Genes

**Figure 4:**
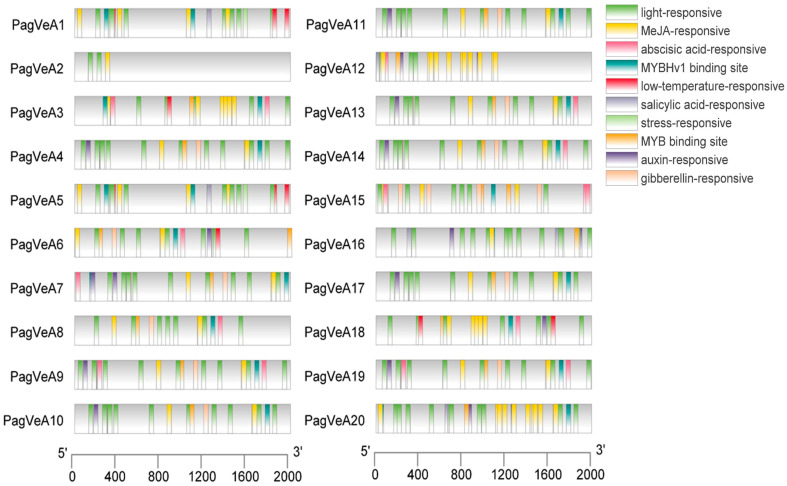
The cis-regulatory elements in the promoter region of the 20 *VeA* genes. The cis-regulatory elements are represented by rectangles in different colors.

Sequence analysis of promoter binding sites is critical for studying the function of transcription factors. The analysis results showed that the promoter positions of these genes contain CAAT-box and MYB binding site, as well as some other cis-acting elements. Their functions are mainly focused on light, phytohormone (MeJA, abscisic acid, salicylic acid, auxin, gibberellin) and stress response processes (Figure 4). Since MYB transcription factors also played a role in the above-mentioned biological processes, this result further confirmed the previous inference that the *VeA* gene was a downstream target gene of MYB transcription factor.

### 2.6. Expression Profiles of VeA Genes in Poplar

**Figure 5:**
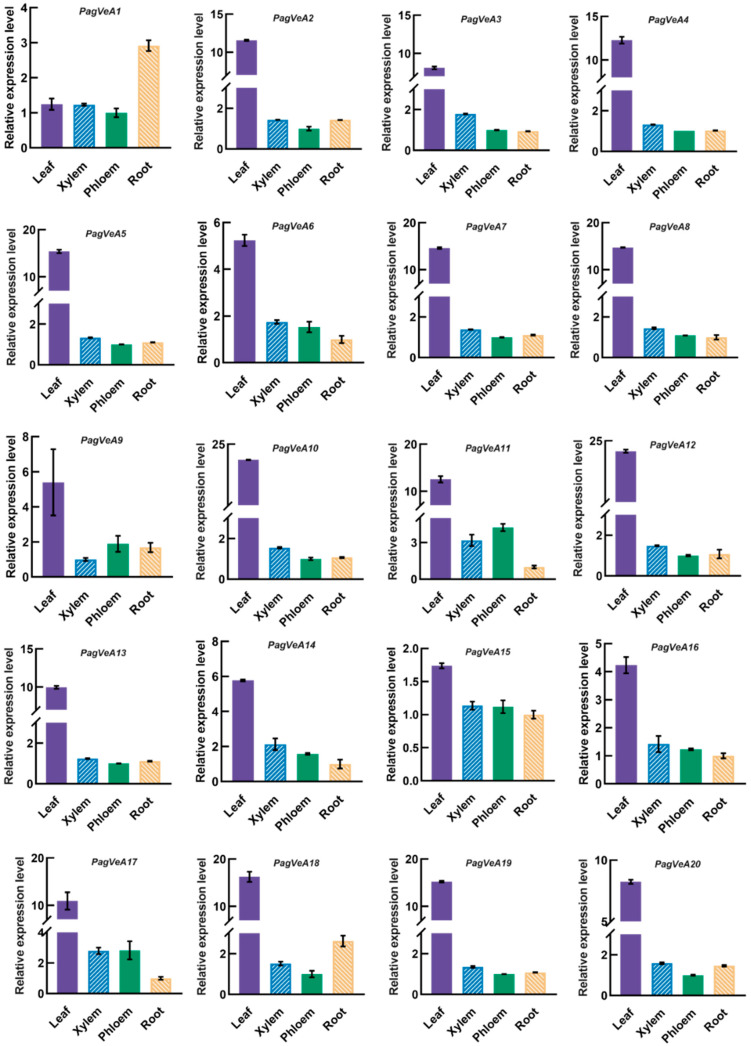
RT-qPCR analysis of the *PagVeA* genes (*PagVeA1-20*) in different tissues (leaf, xylem, phloem, and root) of 3-month-old poplar. Data are presented as means ± SD of three technical replicates.

In order to better understand the functional mechanism of the *VeA* genes in regulating the biosynthesis of secondary metabolites in poplar, we utilized the RT-qPCR method to detect the expression patterns of all members of the *VeA* gene family in various 3-month-old tissues of poplar (leaf, xylem, phloem, and root). The results showed that these genes were expressed in leaf, stem (xylem and phloem), and root, with the highest expression levels observed in the leaf for all *VeA* gene family members except *PagVeA1* (Figure 5). The above gene expression analysis results also suggest that PagVeAs may function mainly in poplar leaves and xylem.

### 2.7. PagVeA3 Directly Regulated by PagMYB128

**Figure 6:**
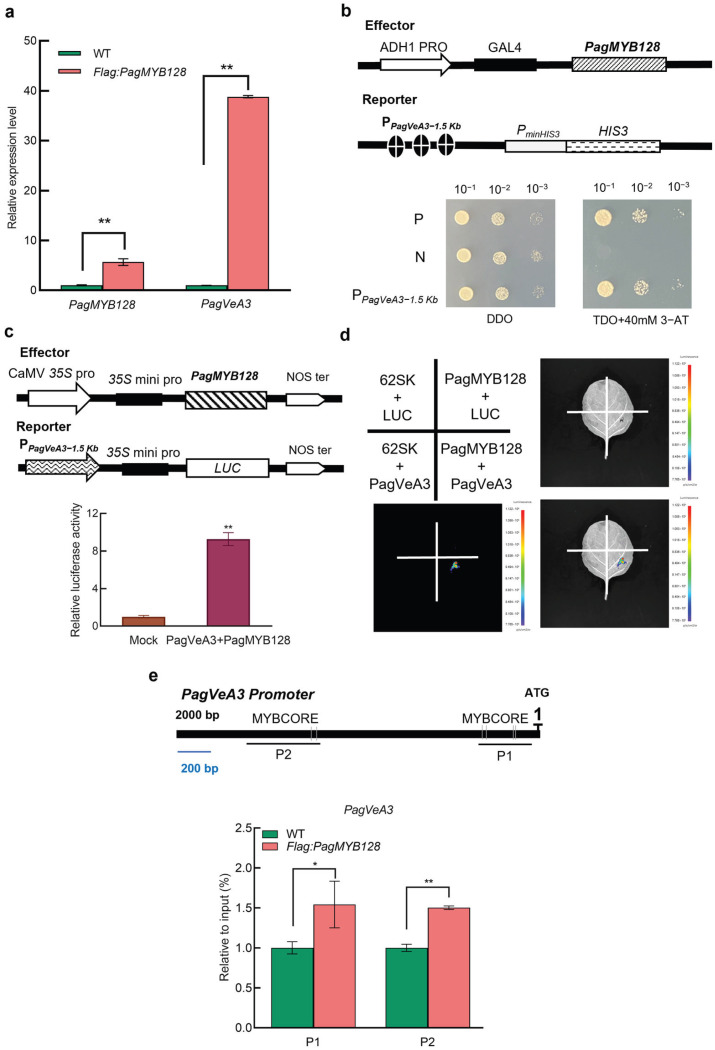
PagMYB128 directly binds to the promoter of *PagVeA3*. (**a**) Transcript abundance of *PagMYB128* and *PagVeA3* in the wild-type (WT) and transiently transformed *Flag:PagMYB128* transgenic plants determined by RT-qPCR. *PagPP2A* was used as an internal control. The values are means ± SEs of three biological replicates. *t*-test: **, *p* < 0.01. (**b**) Y1H assays. The diagram shows the effector and reporter vectors that harbor *PagMYB128* and *PagVeA3* promoter, respectively. Yeast Y187 cells, co-transformed with both effector and reporter constructs, were serially diluted (10^−1^, 10^−2^, 10^−3^) and spotted onto the SDO/-His/-Trp and TDO/-His/-Leu/-Trp medium, supplemented with 40 mM 3-AT. P, positive control (pGAD-53 + p53HIS2); N, negative control (pGADT7-PagMYB128 + p53HIS2). (**c**,**d**) Effector/reporter assays to detect the regulation of *PagVeA3* by PagMYB128. The luciferase activities were measured in tobacco leaves co-transformed of effector (*35S-PagMYB128*) and reporter (*PagVeA3 promoter-LUC*). The empty effector construct was used as mock control. The split-luciferase complementation assay revealed the interaction between PagMYB128 and *PagVeA3* promoter in tobacco leaves. Agroinfiltrated involved construct combinations at the four positions indicated in the upper left. Luciferase activity was only observed in the position representing PagMYB128 + PagVeA3. Data are presented as means ± SD (*n* = 4). Asterisks indicate significant differences (*t*-test) compared to the mock. ** *p* < 0.01. (**e**) ChIP-qPCR assays. In the diagram of the *PagVeA3* promoter region (long thick line), short thin lines show the two fragments (P1 and P2) that contain the MYBCORE motif in the *PagVeA3* promoter, and qPCR primers were designed to amplify these two fragments. ChIP was conducted in wild-type (WT) and *Flag:PagMYB128* transient overexpression plants using anti-FLAG antibodies. The values are means ± SEs of three biological replicates. *t*-test: *, *p* < 0.05; **, *p* < 0.01.

Our previous studies have found that *PagVeA3* may be the downstream target gene of *PagMYB128*. To deeply analyze the mechanism of PagVeA3, we further studied its interaction with PagMYB128. In the *PagMYB128* transient overexpression transgenic plants (*Flag:PagMYB128*), the expression level of *PagVeA3* was significantly increased compared to that in wild-type plants (Figure 6a). This result indicated that *PagVeA3* can be regulated by PagMYB128. In yeast one-hybrid experiment, we found that Y187 yeast strain containing co-transformed *pGADT7-PagMYB128* and *pHIS2-PagVeA3* could grow normally on SDO/-Trp/-Leu and TDO/-Trp/-Leu/-His deficient medium containing 40 mM 3-amino-1,2,4-triazole (3-AT) (Figure 6b). In the effector-reporter assays, effector (35S promoter driven *PagMYB128* gene) and reporter (1.5 kb *PagVeA3* promoter-driven luciferase gene) were co-transformed in tobacco leaves. LUC fluorescence detection exhibited a significant increase when compared to the mock that was transformed with an empty effector construct (Figure 6c). Strong LUC fluorescence signals could be collected under the living imaging instrument (Figure 6d). Previous analysis of the promoter sequences revealed that there were two MYB binding sites (MYBCORE motifs) located within the *PagVeA3* promoter region. We conducted a ChIP-qPCR experiment using WT and *Flag:PagMYB128* transgenic plants. ChIP-qPCR results demonstrated significant enrichments in the P1 and P2 fragments. These results suggested the in vivo binding capacity of PagMYB128 to the promoter region of *PagVeA3* gene (Figure 6e).

## 3. Discussion

This study presented several significant findings through the comprehensive analysis of the specific *VeA* gene family in poplar. Firstly, genome-wide identification suggested 20 putative *VeA* genes in *P. alba* × *P. glandulosa* genome. Phylogenetic analysis demonstrated the evolutionary conservation of PagVeA3 and PagVeA4, indicating their vital roles. The examination of gene structure and conserved motif analysis showed similarities in exon-intron patterns and motifs among genes clustered in the same branch of the phylogenetic tree, affirming their close evolutionary relationships. Promoter binding site analysis unveiled the presence of key regulatory elements associated with light responsiveness, phytohormone response, and stress response, confirming the potential involvement of *VeA* genes in diverse biological processes. Expression profiling through RT-qPCR across different tissues demonstrated ubiquitous expression, with higher levels in leaves and roots. This suggests the potential role of VeA genes in regulating secondary metabolites, particularly in leaves and xylem. 

Some studies have found the existence of *VeA* genes in fungi as well, which influences secondary development and metabolism [40]. Our previous genome annotation file indicated that VeA transcription factors were important in plant developmental process and evolutionary novelty of poplar [4]. To verify the specificity of *PagVeA3*, sequence alignment with *VeA* genes in various fungi was conducted. The low similarity between PagVeA3 and typical VeA proteins in fungi suggested that *PagVeA3* was not an endophytic gene of *P. alba* × *P. glandulosa*. Additionally, comparison with the NCBI database revealed the presence of the conserved region in *P. alba*, further supporting the gene’s significance. However, this gene is currently only found in the *P. alba* and *P. alba* × *P. glandulosa* poplar genomes. On one hand, it may be a gene unique to the *P. alba*, and on the other hand, it could also be attributed to the limitations of the genome sequencing data. Therefore, the specificity of this gene in poplar species requires further in-depth systematic identification and confirmation.

The MYB transcription factors family plays a crucial role in secondary metabolism and response to adversity in plants. PagMYB128 may play an important role in the biosynthesis of plant secondary metabolites such as anthocyanin and lignin. According to the RNA-seq and ChIP-seq data of our research data, we found that PagMYB128 may regulate *PagVeA3*. However, the molecular mechanism of how *PagVeA3*, as a downstream target gene of PagMYB128, affects secondary metabolism in woody plants has not been explained. qRT-PCR results showed that *PagVeA3* was significantly up-regulated in the *PagMYB128* transient overexpressing plants. Using Y1H, effector-reporter assay, and ChIP-qPCR methods, we confirmed that PagMYB128 transcription factor could directly bind to *PagVeA3* promoter. Notably, the direct regulation of *PagVeA3* by PagMYB128 was elucidated through experiments, providing insights into the regulatory network involved in secondary metabolite biosynthesis of poplar. The above research results only provide some clues for the regulation of secondary metabolism by *PagVeAs* genes. The specific functions of the *VeA* gene family may need to be clarified by creating overexpression and knockout mutants and further conducting experiments such as phenotypic observation.

In summary, the comprehensive analysis of the *VeA* gene family in *P. alba* × *P. glandulosa* sheds light on their genomic features, evolutionary relationships, genomic distribution, structural characteristics, and regulatory mechanisms. The findings contribute valuable information for understanding the functional roles of *VeA* transcription factors in poplar, laying the foundation for future research in genetic manipulation and improvement of this economically important tree species.

## 4. Materials and Methods

### 4.1. Plant Materials

Hybrid poplar (*P. alba* × *P. glandulosa*) plants were planted in the greenhouse located at the State Key Laboratory of Tree Genetics and Breeding, Beijing, China. Leaves, xylem, phloem, and roots were collected from 3-month-old plants and stored in liquid nitrogen for RNA extraction of tissue expression-specific analysis. 

Tobacco (*Nicotiana benthamiana*) seeds were seeded into the soil mixed with perlite and placed in the growth chamber (temperature 22 °C, light cycle 16/8 h, relative humidity 70–75%) for one month in a transcription activation experiment.

### 4.2. Gene Cloning and Vector Construction

According to the genome sequences of *P. alba* × *P. glandulosa*, the full-length coding sequence of *PagMYB128* was cloned with the specific primers FLAG-MYB128-F/R in Table S1. The amplified product was inserted into *pBI121-2×35S-3×FLAG* [41] with a CloneExpressII One Step Clone Kit (Vazyme Biotech, Dalian, China) to produce *pBI121-2×35S-3×FLAG:PagMYB128*. The obtained construct was transformed into *Agrobacterium tumefaciens* GV3101. 

### 4.3. Poplar Transient Transformation

Poplar plants were propagated in 1/2 Murashige and Skoog (PhytoTech, Lenexa, KS, USA) solid medium (pH = 5.9), which contained 25 g sucrose and 5.8 g agar. After 30 days, tissue culture seedlings with large, flat leaves and thick, straight stems were selected as the transient transformation materials. The vectors were soaked in the transient transformation of Sultion 1 (1/2 MS medium, 5% [*w/v*] sucrose, 100 μM acetosyringone, 0.01% [*w*/*v*] Tween 20, pH = 5.8) with the addition of *Agrobacterium* at OD_600_ = 0.7–0.8; they were then incubated at 25 °C and 100–130 rpm for 2–3 h until *Agrobacterium* permeated into the tissue culture seedlings. The transiently transformed poplar plants were soaked in Sultion 2 (40 mM CaCl_2_, 3% [*w*/*v*] sucrose, 100 μM acetosyringone, pH = 5.8) for 5 min; we then blotted the water on the filter paper and placed it in 1/2 MS medium for two days. The expression level of *PagMYB128* was identified by RT-qPCR. The transient overexpression transgenic plants of *PagMYB128:FLAG* were collected in liquid nitrogen for ChIP experiments. 

### 4.4. Phylogenetic Tree, Contigs Location, Conserved Motifs, and Promoter Prediction Assays

The contigs localization of these *VeA* genes was based on the *P. alba* × *P. glandulosa* genome annotation file [4]. MEGA7.0 software was used to construct the phylogenetic tree by the muscle and neighbor-joining (NJ) method with a bootstrap value of 1000. The chromosome physical positions of these genes were displayed by TBtools, and the gene structures were displayed by the gene structure display server. MEME tools were used to predict the conserved motifs of candidate protein sequences. The maximum number of motifs was set to 15, and other parameters retained their default settings. Using the Gff3 Sequences Extractor program in TBtools to upload the “84K.genome.gff3” file, we set the Up Stream Bases parameter to 2000, obtaining the “84K.promoter.fa” file after running. Then, we uploaded the “84K.promoter.fa” and *PagVeA* ID list through the Quick Fasta Extractor program list, finally obtaining the promoter sequence of the *PagVeA* family. The 2000 bp upstream of the transcription initiation site of the *VeAs* gene was predicted by the PlantCare database to determine the putative cis-regulatory elements.

### 4.5. The Naming of VeA Family Genes

All the identified *VeA* genes were named in a consistent pattern based on their phylogenetic relationships and chromosome location information. Each gene was named after the abbreviation *Pag* (*P. alba* × *P. glandulosa*) of species name. Genes belonging to one genome but located at different chromosome positions were consecutively numbered.

### 4.6. RT-qPCR

The total RNAs were extracted from different tissues (leaves, xylem, phloem, and roots) of 3-month-old poplar using the RNA Easy-Fast Plant Tissue Kit (TIANGEN, Beijing, China). HiFiScript cDNA Synthesis Kit (CWBIO, Taizhou, China) was used to generate cDNA. UltraSYBR Mixture (CWBIO, Taizhou, China) kit was used for RT-qPCR analysis. *PagPP2A* [41] was used as an internal reference gene [42]. The primers of all genes were listed in Table S1. The results were analyzed by 2^−∆∆Ct^ method.

### 4.7. Yeast One-Hybrid (Y1H) Assays

Using AD-MYB128-F/R and pHIS2-VeA3-F/R primers, cDNA and genomic DNA of target genes were cloned into *pGADT7-Rec2* and *pHIS2* vectors with Phanta Max Super-Fidelity DNA polymerase (Vazyme Biotech, DaLing, China) and ClonExpressII one-step cloning Kit (Vazyme Biotech, DaLing, China), respectively. The recombinant vector was transformed into yeast strain Y187 by PEG/LiAC method. The transformants were screened for positive clones on SD/-Trp-Leu medium and then transferred to TDO/-Trp-Leu-His medium containing 50 mm 3-amino-1,2,4-triazole (3-AT). The co-transformed Y187 cells with *pHIS2-53* and *pGADT7-53* were used as positive control, and *pHIS2-53* and *pGADT7-MYB128* were used as negative control. All primers used for Y1H assays are listed in Table S1.

### 4.8. Transient Transcriptional Activation Assay

This experiment was carried out with previously reported methods [43]. Double luciferase report transient transactivation test was performed. The CDS of *PagMYB128* was inserted into *pGreenII 62-SK* effector, and the 1.5 kb upstream sequence of *PagVeA3* promoter was inserted into *pGreenII 0800-LUC* reporting vector. The resulting effectors and reporter constructs were transformed into *Agrobacterium tumefaciens* by heat shock method and then co-transformed into one-month-old tobacco (*N. benthamiana*) leaves. The luciferase activities of two independent tobacco plants were determined by the double luciferase reporting system (Promega, GloMax 20/20 Lighteter, Madison, WI, USA), and three biological repeated tests were carried out. The plant living imaging system 843 (PlantView100, BLT, Guangzhou, China) was used to collect LUC signals. All primers used for transcriptional activation assay are listed in Table S1.

### 4.9. ChIP-qPCR

ChIP immunoprecipitation was performed according to the method previously described [44]. Wild-type and *PagMYB128:FLAG* transgenic plants were used as experimental materials, and anti-FLAG antibodies (Merck, Darmstadt, Germany) were used for IP. Three biological replicates were carried out, and the purified ChIP-DNA of the replicates were used for qPCR analysis. The *PagPP2A* gene [42] was used as internal reference control. All primers used for ChIP and ChIP-qPCR are listed in Table S1.

## 5. Conclusions

Based on phylogenetics, genomic distribution, gene structure and conserved motif, promoter binding site, and expression profiles analysis, this study comprehensively and systematically identified 20 *VeA* transcription factors in *P. alba* × *P. glandulosa*. In addition, Y1H, effector-reporter, and ChIP-qPCR results confirmed that PagMYB128 could directly bind to *PagVeA3* promoter region, thereby playing an important role in regulating secondary metabolism in poplar. Our results provide a basis for the biological function and regulatory mechanism of *VeA* gene family in poplar.

## Figures and Tables

**Table 1:** Sequences feature of *VeA* genes in *P. alba* × *P. glandulosa*.

Gene ID	Length (bp)	Length (aa)	PI	MW (kDa)	Subcellular Location
Pag_A_036054-RA	1923	640	11.99	66.98	Nucleus
Pag_A_036057-RA	1962	653	12.15	68.15	Nucleus
Pag_A_037007-RA	1986	661	12.15	69.07	Nucleus
Pag_A_037045-RA	1791	596	12.34	62.84	Nucleus
Pag_A_037060-RA	1962	653	12.2	69.04	Nucleus
Pag_A_036660-RA	1929	642	12.3	67.66	Nucleus
Pag_A_037637-RA	1839	612	12.28	64.73	Nucleus
Pag_A_036891-RA	1737	578	12.2	60.9	Nucleus
Pag_A_037554-RA	1767	588	12.08	61.8	Nucleus
Pag_A_035083-RA	2379	792	12.14	83.6	Nucleus
Pag_A_036864-RA	1656	551	12.34	58.62	Nucleus
Pag_A_035064-RA	1656	551	12.34	58.58	Nucleus
Pag_A_037208-RA	1650	549	12.21	58.46	Nucleus
Pag_A_036525-RA	1902	633	12.08	66.94	Nucleus
Pag_A_036382-RA	1191	396	12.67	41.73	Nucleus
Pag_A_037547-RA	1848	615	12.26	65.9	Nucleus
Pag_A_036429-RA	990	329	12.13	35.16	Nucleus
Pag_A_037543-RA	1881	626	12.38	66.15	Nucleus
Pag_A_036646-RA	1836	611	12.1	64.32	Nucleus
Pag_A_037878-RA	1098	365	12.47	38.2	Nucleus

## Data Availability

The RNA-seq and ChIP-seq data have been deposited in the National Center for Biotechnology Information Sequence Read Archive database under accession numbers PRJNA1064460 and PRJNA1056053.

## Supplementary Materials

The following supporting information can be downloaded at: https://www.mdpi.com/article/10.3390/ijms25073926/s1.
