# Supplementary material for: Genome-Wide Identification and Expression Profiling of Velvet Complex Transcription Factors in Populus alba × Populus glandulosa
Source: Int J Mol Sci. 2024 Mar 31;25(7):3926. doi: 10.3390/ijms25073926 (PMC11011700; doi:10.3390/ijms25073926)
Supplement: Supplementary file 1 [file ijms-25-03926-s001.zip › Supplementary Figures S1-S2.pdf]

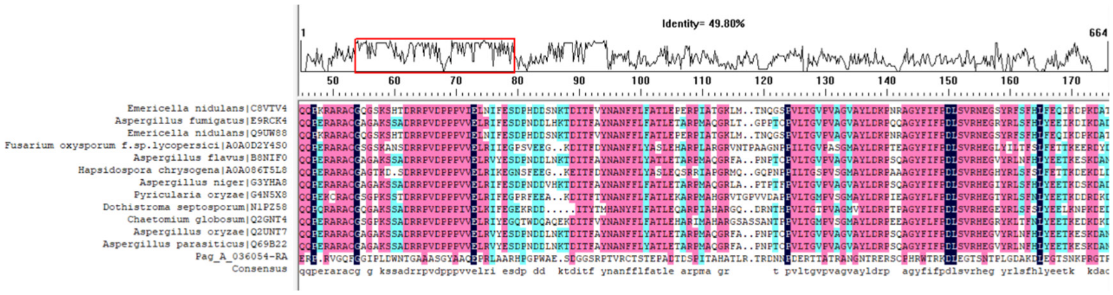


**Supplementary Figure S1.** Results of multi-sequence alignment of Pag_A_036054-RA with a different fungus named VeA. Fungal sequences are named after their genus names and UniProtKB numbers, with the Pag_A_036054-RA protein sequence located at the bottom.

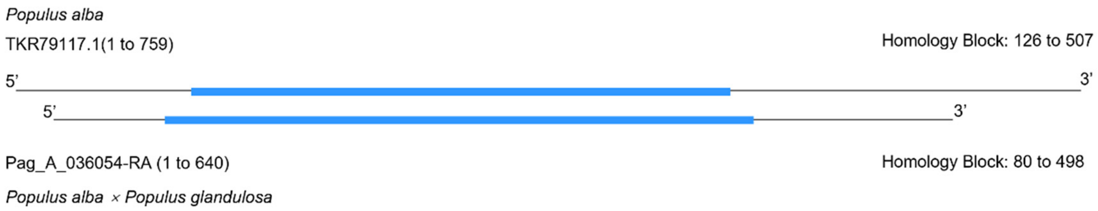


**Supplementary Figure S2.** The BLASTp result of Pag_A_036054-RA in *P. alba*. The similarity between the amino acids from 126th to 507th in the protein sequence of TKR79117.1 in *P. alba* and the amino acids from 80th to 498th in the protein sequence of Pag_A_036054-RA *P. alba* × *P. glandulosa* is as high as 89%.
